# Glucose metabolic reprogramming and its therapeutic potential in obesity-associated endometrial cancer

**DOI:** 10.1186/s12967-022-03851-4

**Published:** 2023-02-07

**Authors:** Pengzhu Huang, Xiangqin Fan, Hongfei Yu, Kaiwen Zhang, Huanrong Li, Yingmei Wang, Fengxia Xue

**Affiliations:** 1 Department of Obstetrics and Gynecology, Tianjin Medical University General Hospital, 154 Anshan Road, Heping District, Tianjin, 300052 China; 2 Tianjin Key Laboratory of Female Reproductive Health and Eugenics, Tianjin Medical University General Hospital, Tianjin, China

**Keywords:** Endometrial cancer, Metabolic syndrome, Glucose metabolic reprogramming, Glycolysis, Mitochondria

## Abstract

Endometrial cancer (EC) is a common gynecological cancer that endangers women health. Although substantial progresses of EC management have been achieved in recent years, the incidence of EC still remains high. Obesity has been a common phenomenon worldwide that increases the risk of EC. However, the mechanism associating obesity and EC has not been fully understood. Metabolic reprogramming as a remarkable characteristic of EC is currently emerging. As the primary factor of metabolic syndrome, obesity promotes insulin resistance, hyperinsulinemia and hyperglycaemia. This metabolic disorder remodels systemic status, which increases EC risk and is related with poor prognosis. Glucose metabolism in EC cells is complex and mediated by glycolysis and mitochondria to ensure energy requirement. Factors that affect glucose metabolism may have an impact on EC initiation and progression. In this study, we review the glucose metabolic reprogramming of EC not only systemic metabolism but also inherent tumor cell metabolism. In particular, the role of glucose metabolic regulation in malignant properties of EC will be focused. Understanding of metabolic profile and glucose metabolism-associated regulation mechanism in EC may provide novel perspective for treatment.

## Introduction

Endometrial cancer (EC) is one of the most common gynecologic malignancies worldwide. The incidence of EC is rising in recent years especially in young women, which deeply influences reproductive health and imposes heavy social burden. The American Cancer Society estimates that in 2022, there would be 65,950 new cases and 12,550 deaths with EC in the United States, making it the fourth most common cancer and the sixth most common causes of cancer death of women [1]. As the most common subtype of EC, endometrioid carcinoma (type I) is hormone-receptor-positive with less-aggressive characteristic and better prognosis compared with type II. Multiple factors contribute to the development of EC including obesity, metabolic dysregulation, excess estrogen and genetic predisposition [2]. Standard treatment for EC is primary hysterectomy and bilateral salpingo-oophorectomy, whereas lymph node dissection, adjuvant or targeted treatment is conducted according to the clinical pathological and molecular characteristic. Progestin-based conservative treatment of EC is viable for those women who wish to preserve fertility with well-differentiated, clinical stage 1 A, endometrioid EC [3]. Although substantial advances of EC management have been made in recent years, the incidence of EC remains high and our understanding of EC is limited. Filling the gap in this field would contribute to develop novel therapeutic strategies and to optimize the treatment for EC.

Metabolic reprogramming includes the rewiring of glucose, lipid and amino acid, which is essential for cancer cells growth and metastasis. As one of the most essential generation modes of bioenergy, glucose metabolism has been studied extensively especially its role in cancer. This metabolic behavior supplies energy required for the biological events and acts as the initiator for malignant events of cancer cells. Cancer metabolic process is modulated by not only intrinsic cellular metabolism but also systemic metabolic condition [4]. Cancer cells regulate the metabolic mode through diverse pathways to meet the energy demand for the biosynthesis. As one of the most commonly abnormal activated pathways in EC [5], PI3K/Akt signaling network induces tumorigenesis and has various downstream impacts on the metabolic process including glucose utilization, lipid and nucleotide synthesis, which is through not only direct phosphorylating the metabolic enzymes but also regulating them via transcription factors responsible for the gene expression [6]. The tumor microenvironment (TME) forms a specific metabolic environment for cancer cells because of nutrient availability, which causes metabolic dependencies of cancer cells [7]. Increasing evidences have shown that cytokines like interleukin (IL)-2, IL-6, IL-10 and tumor necrosis factorα (TNF-α) act on the downstream mediators that affects cellular metabolism and functions [8–10]. Aberrant increase of serum ILs is associated with more aggressive pathologic features of EC [11, 12], which may partly be caused by the metabolic alteration. Impaired glycolysis and enhanced mitochondrial activity are associated with endometrial hyperplasia in an estrogen receptor (ER) α-dependent way in polycystic ovarian syndrome (PCOS) that might be an early hallmark of EC [13].

**Fig. 1:**
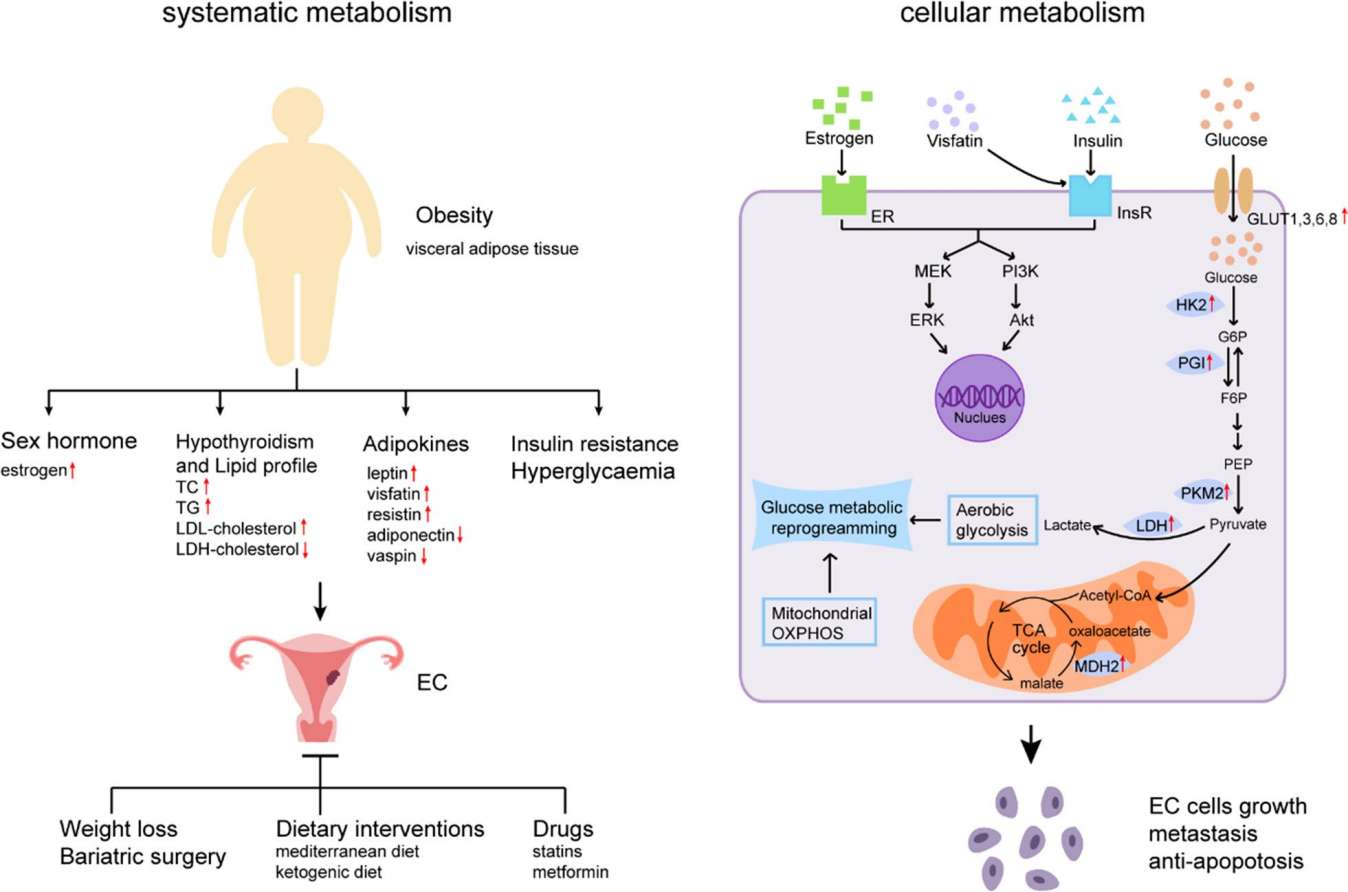
Glucose metabolic reprogramming in obesity-associated endometrial cancer (EC). Obesity with increased visceral adipose tissue is a remarkable feature of EC and contributes to its tumorigenesis and progression through regulation of both systematic metabolism and cellular metabolism. Obesity leads to multiple risk factors of EC including excessive estrogen, abnormal lipid profile, dysregulated adipokines, insulin resistance and hyperglycaemia. Dyslipidemia exists in EC patients especially in those combined with hypothyroidism, including increased level of total cholesterol (TC), low triglycerides (TG), density lipoprotein (LDL)-cholesterol, and reduced level of high-density lipoprotein (HDL)-cholesterol. Dysregulated adipokines are closely associated with insulin resistance, which is primary pathophysiological basis of EC. Insulin directly or synergistically with estrogen promotes malignancy of EC cells through downstream PI3K/Akt and MEK/ERK signaling pathways. Visfatin has similar effects through activation of insulin receptor (InsR) and its substrate. Glucose enters into EC cells through glucose transporters (GLUT) and rewires cellular glucose metabolism. Increased expression or activity of glycolytic enzymes including hexokinase 2 (HK2), phosphoglucose isomerase (PGI), pyruvate kinase isozymes M2 (PKM2), lactate dehydrogenase (LDH) and mitochondrial enzyme malate dehydrogenase 2 (MDH2) in EC tissues may cause metabolic reprogramming which regulates EC cellular behavior

Here, we review recent research advances about the role of glucose metabolism in obesity-associated EC. The role of obesity in EC and its associated systematic and cellular glucose metabolism would be focused. Role of the glucose metabolic regulation in EC tumorigenesis would be discussed in particular (Fig. 1). These perceptions about glucose metabolism would supply a deep understating towards the pathogenic mechanism of EC, thus may provide potentially new therapeutic targets.

### Metabolic syndrome and EC

Metabolic syndrome is a series of pathological condition characterized by metabolic disorder of glucose, lipid and protein, including diabetes mellitus, obesity, hypertension, cardiovascular and cerebrovascular diseases. EC is closely associated with metabolic syndrome that plays an important role in its development and influence the prognosis of patients. A meta-analysis of 6 studies including 17,772 EC patients and 150,371 healthy controls have shown that women with metabolic syndrome have higher risk of EC (OR 1.62, 95% CI 1.26–2.07) [14]. A case-control study comprising 16,323 EC cases and 100,751 controls have found that the component factor of metabolic syndrome is also correlated with higher risk of EC: overweight/obesity (OR 1.95, 95% CI 1.80–2.11), impaired fasting glucose (OR 1.36, 95% CI 1.30–1.43), hypertension (OR 1.31, 95% CI 1.25–1.36) and high triglycerides (OR 1.13, 95% CI 1.08–1.18) [15]. Metabolic syndrome is closely associated with more aggressive clinicopathological features of EC. The proportion of grade 2–3, stage II–IV, lymph node metastasis, lymph-vascular space invasion or deep-myometrial infiltration is higher in EC patients with metabolic syndrome than those without [16]. A prospective cohort study has reported that metabolic syndrome is associated with worse overall survival (HR 1.98, 95%CI 1.07–3.67) of EC patients [17]. These studies indicate that metabolic syndrome plays a critical role in the development and progression of EC in an epidemiological sense. However, the mechanism of metabolic syndrome in malignancy of EC is still not totally clear. Figuring out how components of metabolic syndrome work in EC may help us understand this causal relation more comprehensively.

### Obesity and EC

As the factor of metabolic syndrome, obesity has been reported to be a remarkable risk factor of EC. Compared to women with body mass index (BMI) of 18.5 to 25 kg/m^2^, symptomatic premenopausal women with BMI more than 25, 30 and 40 were at 3.85, 5.25 and 19.79 times higher risk of EC, respectively [18]. In addition to BMI, other obesity-associated measurements including weight, weight gain, waist circumference, waist-to-hip ratio and hips circumference were correlated with increased risk of EC [19]. The impact of obesity on EC varies at different time points in life course. Compared to early life, obesity in adulthood has a stronger association of EC risk [20, 21]. Every 10-year increase of the adult overweight duration (BMI ≥ 25 kg/m^2^) has been reported to be correlated with 17% higher risk of postmenopausal EC [22]. Besides baseline BMI, weight gain (per one kg/year increase) during adulthood was also associated with EC (HR 1.14; 95% CI 1.05–1.23) [23]. In addition to the impact of obesity on EC incidence, it also affects the recurrence and survival of EC survivors. One meta-analysis including 46 studies have found that higher BMI (≥ 30 kg/m^2^) at the diagnosis of EC was correlated with increased recurrence (HR 1.28, 95%CI 1.06–1.56) and all-cause mortality (HR 1.34, 95%CI 1.12–1.59) [24].

Obesity contributes to EC tumorigenesis that may be caused by the dysregulation of hormonal and metabolic homeostasis. Adipose tissue is one important source of estrogen since peripheral aromatization process that converts androgen to estrogen which could cause high level of estrogen in obese women; excessive estrogen condition links obesity and EC development especially in postmenopausal women with increased visceral adipose tissue [25]. Nine BMI-associated single nucleotide polymorphisms (SNPs) variants identified by genome-wide association studies (GWAS) have been found to be related with EC risk, which represent loci in genes including SEC16B/RASAL2, TMEM18, MSRA, SOX6, MTCH2, FTO and MC4R [26]. FTO polymorphisms rs9939609 was reported to be associated with not only metabolic changes in PCOS [27] but also risk of EC [28]. As the m6A demethylase, obesity-associated gene FTO has been reported to rewire glycolytic metabolism through elevation of transcription factors c-Jun, JunB, and C/EBPβ in cancer cells, which further regulates the immune escape and promotes tumor growth [29]. β-estradiol (E2) is the bridge that links obesity and EC progression. E2 enhances FTO expression in EC through PI3K/Akt and MPAK signaling pathways, which then promotes proliferation and invasion of EC cells [30]. Therefore, obesity as the driving factor disrupts systematic metabolism and promotes to EC development. Targeting on obesity may be a promising target for EC therapy.

### Obesity and other metabolic diseases in EC

Obesity is commonly associated with endocrine and metabolic disorders like thyroid dysfunctions and cholesterol imbalances. Although it is still unknown whether obesity is the origin or complications of these diseases, current evidences indicate their synergistical involvement in the malignancy and poor outcomes of EC. Hypothyroidism, characterized by increased circulating thyroid-stimulating hormone (TSH) level, is prevalent in people with obesity [31]. One meta-analysis including 22 studies evaluating the relationship of obesity and thyroid diseases have suggested that obesity is associated increased risk of hypothyroidism (OR 1.86, 95% CI 1.63–2.11) [32]. Hypothyroidism is one common morbidity of EC and not only directly affects the endometrium but also interacts with EC-associated risk factors such as metabolic syndrome, PCOS and infertility [33]. One study investigating the impaction of thyroid function on the prognosis of EC patients have found that higher level of pre-therapeutic serum TSH (> 2.5 mU/L) was associated with poor disease-specific survival (HR 2.7, 95%CI 1.1–6.7) [34]. However, one prospective cohort study enrolling 331 EC patients with median follow-up of 35 months have found that women with the history of hypothyroidism had improved overall survival (adjusted HR 0.22, 95%CI 0.06–0.74), cancer-specific survival (adjusted HR 0.21, 95%CI 0.05–0.98) and recurrence-free survival (adjusted HR 0.17, 95%CI 0.04–0.77) than those without [35]. Dyslipidemia is one of the most common metabolic disorders in hypothyroidism including increased level of total cholesterol (TC), low density lipoprotein (LDL), and triglycerides (TG) were significantly higher and reduced high-density lipoprotein (HDL) level compared to euthyroid individuals [36]. Both decreased thyroid hormone (TH) and increased TSH act on lipid metabolism through regulation of production and clearance of cholesterol in the liver [37]. Dyslipidemia was found in EC patients with increased level of TC, TG, LDL-cholesterol and reduced level of HDL-cholesterol [38]. Pretreatment TG/HDL-cholesterol ratio was increased in EC patients compared to controls and high TG/HDL-cholesterol ratio (≥ 1.52) in postmenopausal women could be used as an independent predictor of EC (OR 4.123, 95%CI 2.633–6.457) [39].

### Obesity and adipokines in EC

As an endocrine organ, the adipose tissue secretes abundant biological active factors such as adipokines that have a local or systematic impact on multiple physiological process such as lipid and glucose metabolism and insulin sensitivity. Higher levels of leptin, visfatin and resistin have been found in EC patients, which is associated with malignant clinicopathology and poor prognosis [40–43]. As shown in Table 1, these adipocytokines are involved in insulin resistance which may link obesity and its associated malignancy [44–46]. However, several adipokines like adiponectin and vaspin may act as the protective factor against EC through their roles of insulin sensitizing [44, 47]. Various adipocytokines have direct effects on the proliferation and metastasis of EC cells that impacts the risk and prognosis of EC patients [48]. Among them, visfatin is a kind of insulin-like adipokine that plays a crucial role in the metabolism-associated disease especially EC. Emerging evidences in recent decades have shown increased levels of visfatin in overweight/obesity, type 2 diabetes mellitus, metabolic syndrome, cardiovascular diseases and cancers [45, 49–51]. Besides, circulating visfatin level manifested a positive association with insulin resistance [45], which further supports the role of glucose metabolic dysregulation in obesity-associated diseases. Mechanically, visfatin activates insulin receptor (IR) and insulin receptor substrate (IRS)1/2 of EC cells to enhance their proliferation and inhibit apoptosis through PI3K/Akt and MAPK/ERK signaling pathways [52].

### Obesity and glucose metabolism in EC

#### Insulin resistance

Obesity promotes inflammation and immune responses in adipose tissue, leading to obesity-associated metabolic dysfunction and insulin resistance locally or systematically [53]. Insulin resistance, as the significant basis of metabolic syndrome, closely links obesity with EC. The prevalence of insulin resistance in EC patients is high as reported to be 66.7% in one prospective study [54]. However, increased BMI is significantly associated with higher risk of insulin resistance and in those women with BMI of 30 kg/m^2^ or greater, this proportion reaches up to 84% [54]. Insensitivity of target organs to insulin following insulin resistance results in hyperinsulinemia, impaired fasting glucose and could even develop into diabetes mellitus in severe cases. As the pathophysiological basis of metabolic syndrome, insulin resistance is closely correlated with EC. In one meta-analysis of 13 studies including 1562 EC patients and 2526 controls, fasting insulin level was significantly higher in women with EC [55]. Hyperinsulinemia is associated with not only disordered proliferative endometrium as well as endometrial hyperplasia but also type I EC, indicating that it is involved in the development of endometrial hyperplastic lesions and EC [56]. Xue et al. [57, 58] found that increased serum insulin level was the independent risk factors for type I EC. Insulin resistance also indicates worse prognosis of EC patients. Wang et al. [59] have reported that insulin resistance (OR 9.5, 95% CI 3.3–27.0), metabolic syndrome (OR 4.9, 95% CI 1.5–15.5), condition of both insulin resistance and metabolic syndrome (OR 21.0, 95% CI 4.8–92.7) is significantly associated with the recurrence in patients with atypical endometrial hyperplasia (AEH) and early EC, which may help predict the prognosis and optimize the therapeutic strategies for patients with fertility-sparing treatment. In terms of molecular mechanism, insulin is involved in the progression of EC directly or synergistically with other factors. The insulin receptor (InsR) and InsR substrate (IRS)-1 were found to be activated in EC tissues, which was associated with elevated serum insulin in EC [60]. Insulin promotes EC cells proliferation, anti-apoptosis and invasion through PI3K/Akt and MEK/ERK pathway [60, 61]. Insulin and estrogen synergistically promote type 1 EC progression through activating ER-α and InsR-β respectively and their downstream signaling pathways in a crosstalk way [58, 62]. The estrogen sensitivity of EC cells could also be regulated in an insulin-dependent way. Insulin promotes estradiol-driven EC cells proliferation via up-regulating the expression of Ten-eleven-translocation 1 (TET1), which is a DNA hydroxymethylase that could enhance G-protein-coupled estrogen receptor (GPER) expression [63]. GPER expression is strongly increased in tissues from EC patients with insulin resistance and also positively correlates with TET1 expression in EC tissues, which further indicate the role of insulin in the progression of EC mediated by TET1-driven GPER expression [63].

#### Hyperglycaemia

Since obesity is a main cause of insulin resistance, its subsequent hyperglycaemia furthers involve the development of obesity-associated diseases [64]. Epidemiological studies have shown that hyperglycemia-correlated status increase the incidence of EC, suggesting that there may be a causal relationship between dysregulation of the blood glucose and EC. One Swedish prospective study containing 230,737 women with average follow-up of 11.7 years found that a total of 1070 cases developed EC and increased risk of EC was related to diabetes (HR 1.46, 95% CI 1.09–1.96) and impaired glucose metabolism (HR 1.41, 95% CI 1.08–1.85) [65]. One case-control study including 16,323 patients with EC and 100,751 controls also found that EC risk was associated with impaired fasting glucose (OR 1.36, 95% CI 1.30–1.43) [15]. Liao et al. [66] included 17 prospective studies and 12 retrospective studies for the meta-analysis and confirmed the association between diabetes mellitus and increased risk of EC (RR 1.89, 95%CI 1.46–2.45). Besides, long-term elevated blood glucose may also increase EC risk. Travier et al. [67] found that elevated risk of EC was associated with moderately increase of glycosylated hemoglobin (HbA(1c)) (6–6.9%) (HR 4.05, 95% CI 1.10–14.88) as well as highly increase of HbA(1c) (> or = 7%) (HR 5.07, 95% CI 1.20–21.31).

The role of various concentrations of glucose on EC cells survival has been reported. Han et al. [68] treated EC cells with low glucose (1 mM), normal glucose (5 mM) and high glucose (25 mM) in vitro and found that high glucose promoted malignant phenotype and increase the glucose uptake and glycolytic activity through regulation of AMPK/mTOR/S6 and MAPK pathways. However, how the EC cells uptake high-concentrated glucose and is influenced by hyperglycemia environment? As the essential step of glucose metabolism, the transport of glucose across the plasma membrane mediated by the glucose transporters (GLUT) could achieve the energy supply and regulation of glucose homeostasis. Thus, enhanced expression of specific GLUT may increase the glucose uptake of malignant cells and then promotes tumor progression. In EC tissues, up-regulated GLUT expression has been reported to associate with the clinicopathological features. Increased GLUT3 mRNA level was significantly with elevation of the tumor grade in EC tissues [69]. Compared with benign lesion of endometrium, GLUT1 immunostaining is more remarkable in complex hyperplasia with atypia and in adenocarcinoma, which might be used to distinguish benign and malignant lesions of endometrium [70]. The protein expression of GLUT1 and GLUT8 are higher in uterine papillary serous EC tissues than well-differentiated or poorly-differentiated adenocarcinoma EC tissues [71]. GLUT6 is highly expressed in EC cancerous glandular epithelial cells, whereas not or little expressed in the glandular cells from normal endometrium; suppression of GLUT6 expression could inhibit glycolysis and survival of EC cells in vitro [72].

#### Aerobic glycolysis

Aerobic glycolysis also named the Warburg effect has linked tumor biological events and metabolism since last century. It refers to the phenomenon that tumors prefer to consume glucose and generate lactate even in presence of oxygen, which may be caused by damaged oxidative respiration [73]. Recent studies about the role of glycolysis in EC mainly focus on aberrant expression of glycolytic genes and enzymes. Glycolysis-associated gene or long noncoding RNA signature in EC has been constructed that could supply the survival prediction of EC patients in clinic [74–76]. As the enzyme that catalyzes the first step in glycolysis, hexokinase 2 (HK2) is upregulated in EC that could be used as the predictor of worse prognosis [77]. Pyruvate kinase (PK) catalyzed phosphoenolpyruvate (PEP) to pyruvate, which is the final rate-limiting step of glycolysis. Four tissue-specific isoforms of PK include PKL, PKR, PKM1, and PKM2, of which PKM2 is up-regulated in most cancer cells involving in metabolic reprogramming [78]. PKM2 has been found to be highly expressed in malignant endometrial lesions associating with poor overall survival (HR for death 3.40, 95% CI 1.35–8.56), whereas low expression of PKM2 in normal endometrium [79]. PKM1 expression is relatively lack in precancerous than noncancerous samples in patients initially diagnosed with complex hyperplasia with atypia through endometrial biopsy, indicating that suppressed expression of PKM1 may predict the progression of endometrial complex hyperplasia with atypia to EC [80]. However, whether the switch from PKM1 to PKM2 promotes the tumorigenesis of EC and the mechanism of this metabolic switch still needs further investigation. PK and phosphoglucose isomerase (PGI) have significantly higher activity in EC tissue than in normal endometrium [81]. Lactate dehydrogenase (LDH) converts pyruvate to lactate, which is the last glycolytic step. The activity of LDH in EC tissue is higher compared with normal uterine endometrium and normal uterine myometrium (1.8 and 2.8 fold, respectively) [82].

Regulation of glycolytic enzymes, transporters and metabolite may have an impact on the survival and progression of EC cells. LncRNA SNHG16 regulated by the transcript factor TFAP2A promotes glycolysis and proliferation of EC cells through miR-490-3p/HK2 axis [83]. HK2 upregulated by upstream lncRNA DLEU2 promotes epithelial-to-mesenchymal transition (EMT) and glycolysis in EC by activating FAK/ERK1/2 signaling [77]. Galloflavin, a novel LDH inhibitor, supresses malignant phenotype through impaired glycolytic metabolism and generation of reactive oxygen species (ROS), which might be a promising agent for EC treatment [84]. Aldehyde dehydrogenase (ALDH), as a marker of cancer stem cells, is increased in EC tissues than normal and hyperplasia endometrium and could reflect the prognosis of patients with endometrial hyperplasia [85]. ALDH-dependent GLUT1 upregulation contributes to the activation of glycolysis and survival of cancer stem cells; the synergistic role of inhibition of ALDH or GLUT1 combined with taxane suppresses the tumorigenesis, which provides a new prospect for EC treatment [86]. Fructose-1,6-bisphosphate (F-1,6-BP), one metabolic intermediate of glycolytic pathway, promotes the generation of ROS and P53-dependent death in EC cells [87]. Genes associated with the regulation of glycolysis may affect biological behavior of EC cells. Proviral insertion in murine lymphomas 2 (PIM2) phosphorylates AMPKα1 to inhibit AMPKα1 kinase activity, which enhances glycolysis and tumor growth in EC [88]. Kinesin family member C1 (KIFC1) enhances glycolysis and survival of EC cells through HMGA1/c-myc pathway [89]. Anterior gradient 2 (AGR2) that is overexpressed in EC tissue has been reported to induce glucose uptake, lactate production and expression of glycolytic enzymes LDHA, phosphoglycerate kinase 1 (PGK1) and HK2, which causes subsequent EC progression through MUC1/HIF-1α pathway [90]. Upregulation of histone deacetylase 1 (HDAC1) also contributes to glycolysis and progression in EC [91].

#### Mitochondrial regulation

Although Warburg Effect has been the best-characterized metabolic status in tumor, oxidative phosphorylation (OXPHOS) is not inhibited in all types of cancers. It is currently emerging that some cancers including EC are mitochondrial OXPHOS-dependent for the energy need even with active glycolysis [92]. In some cases, OXPHOS has been found to be associated with the prognosis and progression of cancer. Takahashi et al. [93] conducted the Japanese molecular profiling of EC in 85 patients and found that patients with TP53-inactive ECs had poor prognosis and activated OXPHOS, indicating that targeting OXPHOS pathway may be a potential therapeutic strategy for EC. Compared with other subtypes of EC, serous-like ECs presents with substantially increased mitochondrial DNA (mtDNA) copy number [94]. Besides, TP53 mutations, significantly associated with mtDNA abundance, have been found to be enriched in serous-like ECs [94], which implies that mtDNA may involve in the poor prognosis of ECs especially in those with TP53 mutations. The newly constructed OXPHOS-related signature containing ATP5IF1, COX6B1, FOXP3 and NDUFB11 is a great predictive stool for the prognosis of EC, since patients with low-risk score tend to be more immunogenic so that it could stratify patients with EC into different risk groups and optimize the therapy strategies [95].

Malate dehydrogenase (MDH) which catalyzes malate into oxaloacetate is an important NAD-dependent dehydrogenases in the mitochondrial tricarboxylic acid cycle and regulates the metabolism of cancers. MDH2, the isoform of MDH, has been reported to be involved in the development of PTEN-regulated EC [96]. Clinically, MDH2 is overexpressed in the cytoplasm of EC tissues, with a relation to the EC grade. Mechanically, MDH2, co-localizing with PTEN in the cytoplasm, promotes the proliferation and invasion as well as inhibits the apoptosis of EC cells via the suppression of the PTEN expression. Therefore, MDH2 might lead to the disfunction of PTEN and subsequent of the development of EC. In addition to mitochondrial enzyme, genes that mediates mitochondrial energy metabolism may also affect cancer cell survival. COX7RP, one mitochondrial gene coded by the nuclear DNA, involves in the respiratory supercomplex assembly and mitochondrial respiration. Overexpression of COX7RP has been found in EC tissues and could promotes EC cells growth, stabilizes mitochondrial supercomplex assembly and regulate the metabolism in vitro and in vivo [97].

Multiple ways of mitochondrial biology beyond metabolic reprogramming contributes to tumorigenesis such as mitochondrial biogenesis, fission and fusion dynamics [98]. The peroxisome proliferator-activated receptor gamma coactivator 1α(PGC-1α) is an important regulator of mitochondrial biogenesis and oxidative metabolism. It activates nuclear respiratory factors 1 and 2 (NRF-1 and NRF-2) and thus induces the expression of transcription factor A mitochondrial (TFAM), which regulates the mtDNA replication and transcription [99]. The PGC-1α-dependent pathway of mitochondrial biogenesis is upregulated in type I EC, leading to the 2-fold increase of mtDNA content in EC tissue than the proliferative endometrial tissue [100]. Mitochondria are in the dynamic balance between division and fusion, and the disruption of this homeostasis will affect their functions. Dynamin-related protein 1 (Drp1) phosphorylation is increased in EC cells exposed to high glucose, which enhances mitochondrial fission and thus promotes EC cells proliferation, migration and invasion; Drp1 activation is also increased in tissues from EC patients with diabetes than normal endometrial tissues [101]. Besides intracellular mitochondrial movement, recent evidences have indicated that intercellular mitochondrial transfer through tunnelling nanotubes extracellular vesicles and gap junction channels may have potential effects on recipient cells for maintaining body homeostasis and regulating pathological processes [102]. The gap junction protein connexin 43 (Cx43) mediates mitochondrial transfer and is important for intercellular communications [103]. One study reported that Cx43 was weakly expressed in endometrial hyperplasia and EC tissues, suggesting impaired gap junctional intercellular communication in the carcinogenesis of endometrium [104]. Deletion of Cx43 expression in EC cells has been reported to increase EC cellular migration; DNA hypermethylation of GJA1 encoding the protein Cx43 was found in human EC samples which was associated with obesity and in vitro reversal of DNA methylation enhanced intercellular communication and interactions, implying that targeting the activity of gap junction could be used to prevent against obesity-associated EC [105].

The mutations in the fibroblast growth factor receptor 2 (FGFR2) related to poor prognosis have to be found in 12% EC patients with stage I/II and 17% EC patients with stage III/IV [106], indicating that FGFR2 inhibition might be a potential treatment for EC. Packer et al. [107] found that FGFR inhibitors led to FGFR2-mutant EC cell lines death through mitochondrial disfunction as the mitochondrial depolarization, cytochrome c release and mitochondrial respiration damage. IL-24, known as an antitumor gene, may be a potential gene therapeutic target for EC. Overexpression of IL-24 leads to apoptosis via the mitochondrial intrinsic signaling pathway with increased BAX and Cytochrome C as well as decreased BCL-2, Caspase-9 and Caspase-3 [108].

### Glucose metabolism-targeted therapies for EC

#### Weight loss and bariatric surgery

Weight loss is correlated with lower risk and improved survival of EC. One observational study enrolled 36, 794 postmenopausal women (50 to 79 years old) with body weight measured at baseline and year 3, which found that EC risk was reduced in women with weight loss (change ≥ 5%) (HR 0.71, 95% CI 0.54–0.95), especially in obese women with intentional weight loss (HR 0.44, 95% CI 0.25–0.78) [109]. In 875 EC patients, prediagnosis moderate-to-vigorous intensity physical activity (more than 7 h per week) was associated with lower all-cause-5-year mortality (HR 0.57, 95% CI 0.33–0.98) than never or rare exercise, but this correlation was reduced after adjustment for BMI (HR 0.64, 95% CI 0.37–1.12) [110]. In addition to intentional weight loss and physical activity, bariatric surgery has also been reported to reduce the risk of EC. One meta-analysis of 5 studies evaluating the effect of bariatric surgery on EC risk included 113,032 women underwent bariatric surgery as well as 848,864 controls and found that 462 (1.4%) and 11,997 (1.4%) women developed EC respectively (OR 0.317, 95% CI 0.161–0.627) [111]. However, what mechanism contribute to the decreased EC risk in women with weight loss? Firstly, weight loss is associated with changes of sex hormone levels. A prospective study enrolling 106 women with median BMI of 44.5 has found that bariatric surgery reduces the body weight by 32.7% and estradiol level by 35.5% at 1 year after the surgery [112]. Besides, weight loss could improve glucose homeostasis and suppress insulin resistance [113, 114]. Furthermore, weight loss could change the inflammation status with decreased level of C-reactive protein (CRP) and IL-6 [113].

#### Dietary interventions 

Dietary factors have a significant effect in systematic metabolic process and may influence oncogenesis driven by abnormal metabolism. Recent evidences have indicated that interventions targeting dietary patterns and components may reduce risk and improve outcomes of metabolic-associated diseases. Mediterranean diet is a plant-based diet pattern and characterized by high intake of plant foods (fruits, vegetables, breads, whole grains, beans and nuts) and olive oil, moderate intake of wine especially red wine during meals, minimal intake of processed food [115]. Evidences have indicated that mediterranean diet is beneficial for cardiovascular health with reduced incidence of cardiovascular diseases and risk factors including obesity, hypertension, metabolic syndrome and dyslipidaemia, and is also protective against type 2 diabetes and provides better glycaemic control [115]. Since menopause is correlated with weight gain and redistribution of abdominal adipose tissue, increased visceral fat secretes excessive adipocytokines leading to systematic metabolic disorder; therefore, mediterranean diet is recommended for menopausal women to prevent obesity and its associated diseases [116]. Compared with women with low adherence to the mediterranean diet, women with high adherence have a nearly half reduced risk of EC (OR 0.51, 95% CI 0.39–0.86) [117]. In one pooled analysis of three case-control studies including 1411 EC patients and 3668 controls, mediterranean diet is associated with lower risk of EC and the OR for an increment of one component of the mediterranean diet was 0.84 (95% CI 0.80–0.88), and the association was stronger in older women or those who never use oral contraceptive or hormone replacement therapy [118]. However, in one prospective analysis enrolling 84,415 postmenopausal women, there was no association between mediterranean diet and EC risk during follow-up for 13.3 years (HR 0.98, 95% CI: 0.82–1.17) [119].

Ketogenic diet is another dietary pattern that has attracted extensive attention in recent years. It is characterized by a high-fat, adequate-protein and very-low-carbohydrate diet regimen that stimulates metabolic status of fasting leading to the production of ketone bodies [120]. Emerging evidences have indicated that ketogenic diet may be a potential therapeutic strategy for metabolic disorders including epilepsy, type 2 diabetes, obesity, nonalcoholic fatty liver disease, PCOS as well as cancers through reduction of blood glucose and insulin levels, improving insulin sensitivity and regulation of glucose and fat metabolism [120, 121]. Compared with consuming the American Cancer Society (ACS) diet characterized by high-fiber and lower-fat, adherence to ketogenic diet for 12 weeks in ovarian cancer or EC patients was associated with higher physical function score and does not have serious adverse events or affect serum lipid levels, suggesting that short-term ketogenic diet may be a safe approach of dietary intervention for EC patients [122, 123]. In addition, ketogenic diet was reported to be associated with more reduction of visceral fat mass and lower fasting serum insulin than ACS diet in ovarian cancer or EC patients, further indicating the potential therapeutic role of ketogenic diet in EC [124].

### Pharmacological treatments

#### Statins

Statins, also known as 3-hydroxy-methylglutaryl coenzyme A (HMG-CoA) reductase inhibitors, exert a potent lipid-lowering effect and thus are used to prevent cardiovascular disease [125]. Through inhibition of the rate-limiting enzyme cholesterol biosynthesis HMG-CoA and subsequent conversion into mevalonate, it reduces the production of cholesterol, increases cellular LDL receptor and promotes the clearance of serum LDL-cholesterol [126]. However, emerging evidences have uncovered pleiotropic effects of statins in cardiovascular protection beyond cholesterol-reduction, which may through plaque stabilization and regression, anti-inflammation effects [126]. In addition to the use in cardiovascular diseases, statins have been paid great attention for their anti-cancer effect through inhibition of cholesterol-associated pathways responsible for cancer cells proliferation [127]. In vitro, simvastatin and metformin synergistically inhibit EC cells viability through inhibition of mTOR pathway, which indicate the potential of statins for EC therapy [128]. Epidemiological studies have indicated that long-term use of statins is associated with the reduction of EC risk [129, 130] and mortality [131]. Statin use contributes to better prognosis of EC patients with increased overall survival and disease-specific survival [132]. In EC patients with hyperlipemia and high-risk histology, statins use has significantly improved their overall survival than those not using statins [133]. However, several populational evidences reported contrary results and did not support anti-cancer role of statins against EC [134, 135]. Since there are bias about study population and results assessment in current studies, the effect of statins in EC may need to be re-evaluated. Further studies should focus on the effect of statins for EC patients with different lipid profile and histological subtypes, which could help exactly identify patients who may benefit from statins [136].

#### Metformin

Metformin has been widely used as a significant pre-surgical treatment in EC. Metformin is correlated with the reversion of atypical endometrial hyperplasia to normal endometrium [137]. When combined with megestrol acetate, metformin could achieve better prognosis for patients with atypical endometrial hyperplasia than megestrol acetate alone and thus may be a promising fertility-sparing treatment [138]. Besides, metformin is also associated with the reduction of EC risk and could improve the survival of EC patients [137, 139]. In vitro studies have reported that metformin promotes the apoptosis and autophagy and inhibits the proliferation of EC cells through activation of AMPK and subsequent inhibition of mTOR pathway [140, 141]. The PD-L1 expression of EC cells exposed to metformin was reduced through AMPK activation, which further inhibit EC cells growth [142]. In addition to the direct anti-tumor roles, metformin could also inhibit tumor progression through infiltration of tumor-associated macrophages [143] and promotes the M1-like phenotype of tumor-associated macrophages to supress tumor growth and angiogenesis [144]. However, the response to metformin still varies a lot in different patients. One possible cause to that heterogeneity may be hyperglycaemia and hypoxia, which could lead to the switch to glycolysis and reduced metformin response in EC [145]. Targeting on pyruvate dehydrogenase kinase 1 (PDK1) may help solve this dilemma since it could promote the glycolysis of EC cells exposed to long-term high-concentrated glucose; the combination of PDK1 inhibitor JX06 and metformin could inhibit EC cells growth in persistently hyperglycemic environment, which provides the therapeutic prospect for EC patients complicated with diabetes mellitus [146]. Further preclinical investigations are needed to explicit the effect of metformin and potential adjuvant agents in EC therapy, which could help to identify the optimal treatment strategy for EC patients.

#### Biomaterials-based therapy 

Recent development and application of biomaterials and technologies have broadened our insight of the diagnosis and treatment in various diseases [147, 148]. The limits of conventional therapies for cancers accelerate the development of nanotherapies, which have great therapeutic potential for their distinctive features such as increasing the drug therapeutic efficacy and reducing toxicities, targeted delivery of drugs to specific sites and co-delivery of multiple drugs [149]. Multiple nanotechnology platforms like liposomes, albumin nanoparticle and polymeric micelles combining with therapeutic modalities such as chemotherapy, hyperthermia, radiotherapy, gene or RNA interference and immunotherapy have been applied in clinical stage [149]. The oxidative stress amplifying micellar nanoparticles would be a novel anticancer strategy since it been reported to generate ROS and to suppress antioxidant simultaneously, and thus it could make cancer cells more vulnerable to ROS resulting in increased apoptotic cell death and inhibition of tumor growth [150]. Hyaluronic acid (HA)-based hydrogels have shown great potential in drug delivery and targeted cancer therapy since cellular membrane surface HA receptors like CD44 are upregulated in multiple cancer cells [151]. HA-based nanogels loaded with antitumor drugs could be highly internalized by cancer cells overexpressing CD44 and have great inhibition of tumor growth and metastasis [151]. Highly expression of CD44 was reported to be found in 35.4% of EC patients, which was associated with poor overall survival [152]. In vitro study has repeated that HA-functionalized nanomicelles encapsulating suberoylanilide hydroxamic acid (SAHA), one inhibitor of HDAC, increased SAHA delivery and inhibited growth of EC cells expressing CD44 [153]. The application of biomaterials in metabolic-targeting therapy for cancers is still lacking. Current studies mainly focus on the use of versatile platforms with new technologies to combine both metabolic-improving and chemotherapeutic drugs. The co-delivery of metformin and paclitaxel through the folate-modified pH-sensitive micelles leads to higher cytotoxicity and apoptosis against breast cancer cells as well as increased drug uptake and anti-tumor effect in vivo, which could reduce the toxicity of paclitaxel and may be a promising therapy for cancer [154].

## Conclusion

Substantial progresses have been achieved in exploration of metabolic changes in EC. Metabolic syndrome is closely associated with the development and progression of EC, in which obesity and insulin resistance are key points that involve in the pathogenesis process. Obesity plays a core role in EC, which affects not only systemic metabolism but also inherent tumor cell metabolism. Tumor cells adjust the metabolism to meet energy requirement of cellular survival and functions, which promotes tumorigenesis and progression. In this study, the metabolic characteristic especially distinctive changes in glycolytic pathway and mitochondrial functions in EC have been systematically summarized. The known evidences of obesity-associated adipocytokines, insulin resistance and metabolic alterations as crucial oncogenic factors of EC have enabled therapeutic strategies of EC targeting systematic or cellular metabolic process. Management of metabolic syndrome through weight loss, lipid- and glucose-lowing is an effective method for EC treatment and prevention. Various studies have assessed the role of treatments including bariatric surgery, statins and metformin on EC, revealing great potential for reduction of EC risk and improving the survival of EC patients. The lifestyle interventions like mediterranean diet are beneficial for patients with obesity, metabolic syndrome and diabetes, and thus could be used as a safe and feasible approach for preventing EC.

However, since the metabolic pathways in tumors are highly plastic, metabolism is reprogrammed not only in glucose but also in other cellular component such as lipid and amino acid. Besides, the metabolic interactional networks among tumor cells and surrounding microenvironment form the unique metabolic condition, which may together contribute to the development and malignant progress of EC. Although obesity-associated adipocytokines, insulin resistance and subsequent metabolic pathways has been proven to promote malignancy of EC, studies on crosstalk of cellular glucose and lipid metabolism as well as immune responses to these metabolic alterations are still lacking. Figuring out the exact metabolic dependency and compensation mechanism along with response of immune microenvironment towards metabolic status may provide new targets for EC therapy. Further studies are required to stratify EC patients with different metabolic status in view of available therapy and to identify personalized treatment regimen.

**Table 1:** Effects of biologically active factors on EC

Biologically active factors	Roles in EC	References
Leptin	Involving in insulin resistance	[44]
Resistin	Involving in insulin resistance	[46]
Visfatin	Activation of insulin receptor and its substrate IRS1/2 to promote proliferation and inhibit apoptosis of EC cells through PI3K/Akt and MAPK/ERK pathways	[52]
Insulin	Promoting EC cells proliferation and invasion, inhibiting apoptosis through PI3K/Akt and MEK/ERK pathway; Synergizing with estrogen to promote type 1 EC progression through activating ER-α and InsR-β respectively; Promoting EC progression through upregulation of TET1-driven GPER expression	[60, 61] [58, 62] [63]
Adiponectin	Protecting against EC through insulin sensitizing	[44, 47]
Vaspin	Protecting against EC through insulin sensitizing	[44, 47]

## Data Availability

Not applicable.
